# Supplementary material for: 12-Year Trends in Active School Transport across Four European Countries—Findings from the Health Behaviour in School-Aged Children (HBSC) Study
Source: Int J Environ Res Public Health. 2021 Feb 22;18(4):2118. doi: 10.3390/ijerph18042118 (PMC7926861; doi:10.3390/ijerph18042118)
Supplement: Supplementary file 1 [file ijerph-18-02118-s001.pdf]

Supplementary table 1. Sample size and descriptive statistics of independent variables

Variable	Country	2006	2010	2014	2018	Linear trend*
Survey year (n)	Czech Republic	4474	4206	4827	11236	-
	Norway	4074	3990	2793	2613	-
	Scotland	5720	6274	5340	4563	-
	Wales	4049	4432	4739	14882	-
Female sex (%)	Czech Republic	50.2	51.7	52.6	49.9	0.997
	Norway	48.0	50.4	52.6	51.7	**1.014**
	Scotland	51.2	51.0	50.9	52.5	1.003
	Wales	51.3	50.4	50.4	50.5	0.998
Age group (%)						
13 yrs.	Czech Republic	33.7	33.2	33.7	34.4	0.999
15 yrs.		35.3	35.1	35.6	33.2	.0991
13 yrs.	Norway	34.3	29.4	30.6	25.4	**0.942**
15 yrs.		34.7	31.0	29.7	23.2	**0.934**
13 yrs.	Scotland	35.6	30.7	35.4	33.4	0.977
15 yrs.		36.2	38.4	32.5	29.2	0.959
13 yrs.	Wales	36.3	36.4	33.7	33.1	0.984
15 yrs.		32.0	33.0	35.2	33.1	0.994
Travel time (%)						
<5 min.	Czech Republic	24.6	21.7	18.0	18.4	**0.952**
5-15 min.		51.7	47.1	44.7	47.5	**0.970**
<5 min.	Norway	17.6	18.5	20.3	17.8	1.010
5-15 min.		45.5	45.2	45.3	47.3	1.008
<5 min.	Scotland	20.3	20.1	23.1	22.1	1.012
5-15 min.		44.9	43.9	43.3	43.3	0.999
<5 min.	Wales	16.4	15.3	15.0	11.0	**0.944**
5-15 min.		46.1	44.8	44.4	41.0	**0.967**
Family affluence (M)	Czech Republic	0.27	0.39	0.45	0.45	**0.014**
	Norway	0.57	0.65	0.62	0.61	**0.003**
	Scotland	0.38	0.48	0.53	0.55	**0.014**
	Wales	0.39	0.45	0.57	0.60	**0.018**

Ref. Age group = 11 year olds, Ref. Time to school = >15 min. Family affluence - ridit transformed to a linear score (0–1). *Estimates represent odds ratios with exception of family affluence, which are unstandardized regression coefficients. Estimates in bold = p<.001.
